# Effect of Three Nanoparticles (Se, Si and Cu) on the Bioactive Compounds of Bell Pepper Fruits under Saline Stress

**DOI:** 10.3390/plants10020217

**Published:** 2021-01-23

**Authors:** Yolanda González-García, Claribel Cárdenas-Álvarez, Gregorio Cadenas-Pliego, Adalberto Benavides-Mendoza, Marcelino Cabrera-de-la-Fuente, Alberto Sandoval-Rangel, Jesús Valdés-Reyna, Antonio Juárez-Maldonado

**Affiliations:** 1 Doctorado en Ciencias en Agricultura Protegida, Universidad Autónoma Agraria Antonio Narro, Saltillo, Coahuila 25315, Mexico; yolanda_glezg@hotmail.com; 2 Maestría en Ciencias en Horticultura, Universidad Autónoma Agraria Antonio Narro, Saltillo, Coahuila 25315, Mexico; acardenasmjj@gmail.com; 3 Centro de Investigación en Química Aplicada, Saltillo, Coahuila 25294, Mexico; gregorio.cadenas@ciqa.edu.mx; 4 Departamento de Horticultura, Universidad Autónoma Agraria Antonio Narro, Saltillo, Coahuila 25315, Mexico; adalberto.benavides@uaaan.edu.mx (A.B.-M.); marcelino.cabrera@uaaan.edu.mx (M.C.-d.-l.-F.); alberto.sandoval@uaaan.edu.mx (A.S.-R.); 5 Departamento de Botánica, Universidad Autónoma Agraria Antonio Narro, Saltillo, Coahuila 25315, Mexico; jesus.valdes@uaaan.edu.mx

**Keywords:** nanotechnology, salinity, abiotic stress, antioxidants, *Capsicum annuum* L.

## Abstract

The bell pepper is a vegetable with high antioxidant content, and its consumption is important because it can reduce the risk of certain diseases in humans. Plants can be affected by different types of stress, whether biotic or abiotic. Among the abiotic factors, there is saline stress that affects the metabolism and physiology of plants, which causes damage, decreasing productivity and quality of fruits. The objective of this work was to evaluate the application of selenium, silicon and copper nanoparticles and saline stress on the bioactive compounds of bell pepper fruits. The bell pepper plants were exposed to saline stress (25 mM NaCl and 50 mM) in the nutrient solution throughout the crop cycle. The nanoparticles were applied drenching solution of these to substrate (Se NPs 10 and 50 mg L^−1^, Si NPs 200 and 1000 mg L^−1^, Cu NPs 100 and 500 mg L^−1^). The results show that saline stress reduces chlorophylls, lycopene, and β-carotene in leaves; but increased the activity of some enzymes (e.g., glutathione peroxidase and phenylalanine ammonia lyase, and glutathione). In fruits, saline stress decreased flavonoids and glutathione. The nanoparticles increased chlorophylls, lycopene and glutathione peroxidase activity in the leaves; and ascorbate peroxidase, glutathione peroxidase, catalase and phenylalanine ammonia lyase activity, and also phenols, flavonoids, glutathione, β-carotene, yellow carotenoids in fruits. The application of nanoparticles to bell pepper plants under saline stress is efficient to increase the content of bioactive compounds in fruits.

## 1. Introduction

The consumption of fruits and vegetables is important for a healthy diet, because it contributes to reducing the risks of diseases such as cancer, cardiovascular diseases, among others [1]. The bell pepper (*Capsicum annuum* L.) is a vegetable of great economic importance worldwide and with great acceptance in the market for its organoleptic qualities [2], and also for its high content of bioactive compounds [3]. The bell pepper is high in bioactive compounds such as phenols, flavonoids, carotenoids, capsaicinoids, and vitamin C [4]. The intake of this type of compound is important in human health due to its antioxidant, antitumor and antiviral activities, as well as having some other positive effects such as preventing non-communicable diseases [3].

Due to the economic value and high market demand for bell pepper, there is great interest in increasing the production of this crop. However, like all crops, it is constantly threatened by various abiotic stressors such as soil and water salinity [5]. The problems associated with this stress constitute one of the main limitations in food production, especially in semi-arid and arid regions, which is where this problem is most prevalent [5]. Currently, approximately 20% of the total irrigated lands cultivated in the world are affected by salinity and it is estimated that by 2050 it will exceed 50% [6]. This represents a great problem when producing agricultural crops, since, as a consequence of saline stress, osmotic stress is generated, which limits water access to plants, which produces a series of problems such as loss of turgor and cellular dehydration, ionic toxicity, nutritional imbalance, in addition to increasing the production of reactive oxygen species (ROS) [7]. Among the main ROS are superoxide (O_2_^−^), hydrogen peroxide (H_2_O_2_) and hydroxyl radical (OH^−^), which can affect different structures at the cellular level such as proteins, lipids, DNA and cause cell damage [8,9]. Fortunately, plants have defense mechanisms through which they can survive, since they limit ROS production or inactivate them, avoiding or reducing the damage they may cause [10]. This antioxidant system involves a series of enzymatic and non-enzymatic antioxidant compounds that act against free radicals [11]. Enzyme antioxidants are considered the first line of defense, including superoxide dismutase (SOD), catalase (CAT), and glutathione peroxidase (GPX), among others [9].

In the current era, the use of metallic nanoparticles can be an alternative to mitigate abiotic stress, since numerous beneficial effects on the growth and development of crops have been reported. However, these effects depend on the characteristics of the nanoparticles, such as the type of NPs, shape and size, as well as the plant species and the duration of exposure to NPs [12]. Selenium nanoparticles (Se NPs) have potential antioxidant activity as well as low toxicity due to their redox state of zero (Se^0^) [13]. In the cultivation of peanuts (*Arachis hypogaea* L.) they increased the activity of antioxidant enzymes, as well as non-enzymatic antioxidants [14]. They also improved the photosynthetic capacity, accumulation of proline and total soluble carbohydrates for osmoprotection in the cucumber crop [15]. Silicon nanoparticles (Si NPs) can reduce ROS accumulation and generate greater membrane stability, decrease MDA and increase expression of CAT, SOD and APX genes [16]. Under stress conditions the application of Si NPs can increase the antioxidant content [17], and can reduce the negative effects of water stress [18], moreover it can improve the absorption of some nutrients [19]. Copper nanoparticles (Cu NPs) can decrease the absorption and accumulation of sodium (Na) in tomato leaves, which can help to tolerate saline stress [20]. In addition, Cu NPs stimulate the antioxidant defense system of plants resulting in increased production of enzymatic and non-enzymatic antioxidant compounds [21]. Although there are several studies on the impact of nanoparticles in different crops, there is not enough information about the impact on the content of bioactive compounds in the consumption organs of crops developed under conditions of saline stress. For this reason, the present study aims to evaluate the effect of Se, Si and Cu nanoparticles on the accumulation of bioactive compounds in bell pepper fruits of plants developed under salinity conditions.

## 2. Results

### 2.1. Photosynthetic Pigments

**Figure 1:**
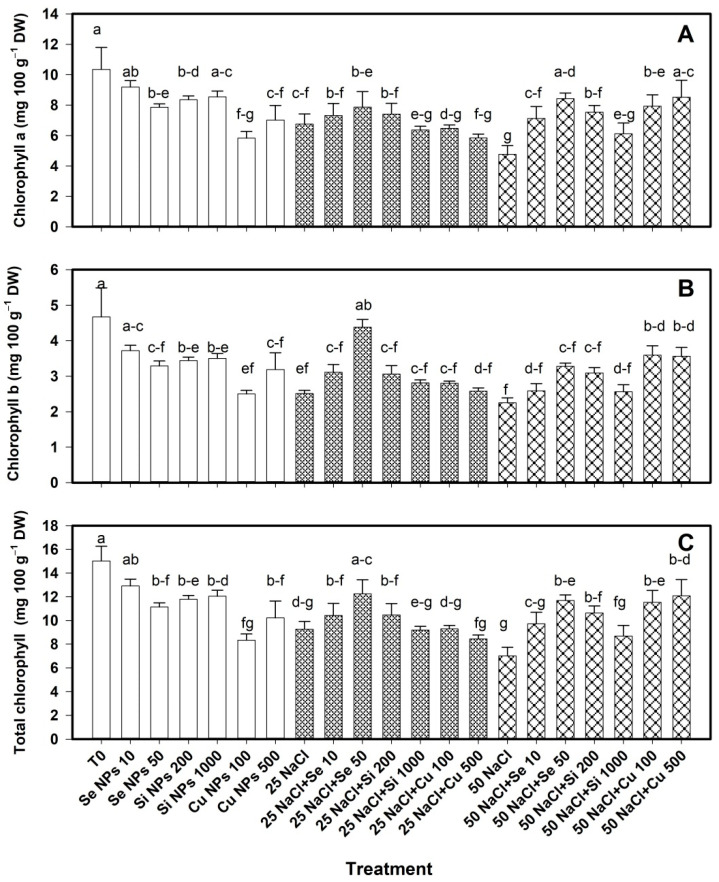
Chlorophyll *a* (**A**), chlorophyll *b* (**B**) and total chlorophyll (**C**) content in leaves of bell pepper plants under saline stress. T0: control; 25 NaCl: 25 mM NaCl; 50 mM NaCl: 50 mM NaCl; NPs: nanoparticles; Numbers represent de quantities of NaCl (mM) or NPs (mg L^−1^) applied. Different letters indicate significant differences between treatments according to Least Significant Difference Fisher’s Test (α = 0.05). n = 5 ± standard error.

The content of photosynthetic pigments was modified with the application of nanoparticles (Figure 1). When the plants were not subjected to saline stress, the chlorophyll content a when with the treatments Se 50, Cu 100 and Cu 500 was decreased by 24%, 44% and 32% respectively compared to the control (Figure 1A). At 25 mM salinity, the treatments had no effect on chlorophyll *a*, however, only salinity at 25 mM decreased the content of this compound by 35%. At 50 mM NaCl, chlorophyll a was increased with all treatments except for Si 1000 (Cu 500 > Se 50 > Cu 100 > Si 200 > Se 10), where the greatest increase was observed with Cu 500 and Se 50 being 79% and 77% more than its control. 50 mM salinity induced the greatest decrease in chlorophyll *a* compared to T0 (−54%).

The chlorophyll *b* content was also modified with the application of nanoparticles (Figure 1B). Without stress due to salinity, with the exception of the Se 10 treatment, all the treatments decreased the content of this compound, the effect being greater with Cu 100 (54% less than T0). At 25 mM salinity, only the Se 50 treatment increased chlorophyll *b* by 75% more than its control. While at 50 mM salinity, it increased with the Cu 100 and Cu 500 treatments by 60% and 58% respectively compared to its control. Chlorophyll *b* is consistently decreased by salinity, the effect being more negative with 50 mM NaCl.

Without saline stress, total chlorophyll decreased with all treatments, except Se 10, the effect being greater with Cu 100, which decreased by 55% compared to T0. At 25 mM salinity, only the Se 50 treatment increased the total chlorophyll content by 32% with respect to its control. At a salinity of 50 mM, total chlorophyll increased with the treatments Cu 500 > Se 50 > Cu 100 > Si 200, in a range of 72–52% (Figure 1C).

**Figure 2:**
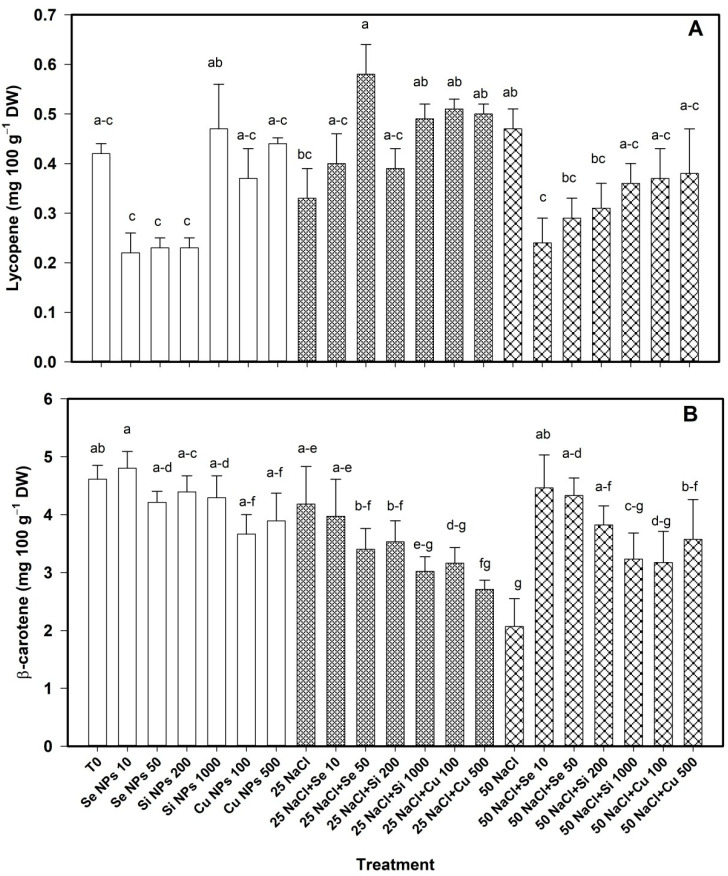
Lycopene (**A**) and β-carotene (**B**) content in the leaves of bell pepper plants under saline stress. T0: control; 25 NaCl: 25 mM NaCl; 50 mM NaCl: 50 mM NaCl; NPs: nanoparticles; Numbers represent de quantities of NaCl (mM) or NPs (mg L^−1^) applied. Different letters indicate significant differences between treatments according to Least Significant Difference Fisher´s Test (α = 0.05). n = 5 ± standard error.

Regarding the accessory pigments, differences were observed in the content of lycopene and β-carotene in the leaves of the pepper plant as a result of the treatments (Figure 2). Lycopene was not modified with the application of nanoparticles when the plants were not subjected to saline stress. However, at 25 mM NaCl this compound was increased with the Se 50 treatment by 76% with respect to its control. At 50 mM NaCl, lycopene decreased by 48% with Se 10 treatment compared to its control. Salinity had no effect on the content of this pigment (Figure 2A).

The β-carotene content was not modified by the treatments when there was no saline stress (Figure 2B). At 25 mM NaCl, the Cu 500 treatment reduced the content of this compound by 35% compared to its control. At 50 mM NaCl, the treatments Se 10 > Se 50 > Si 200 > Cu 500 increased the content of β-carotene in a range of 115–72% with respect to their control.

### 2.2. Antioxidant Activity in Leaves

**Figure 3:**
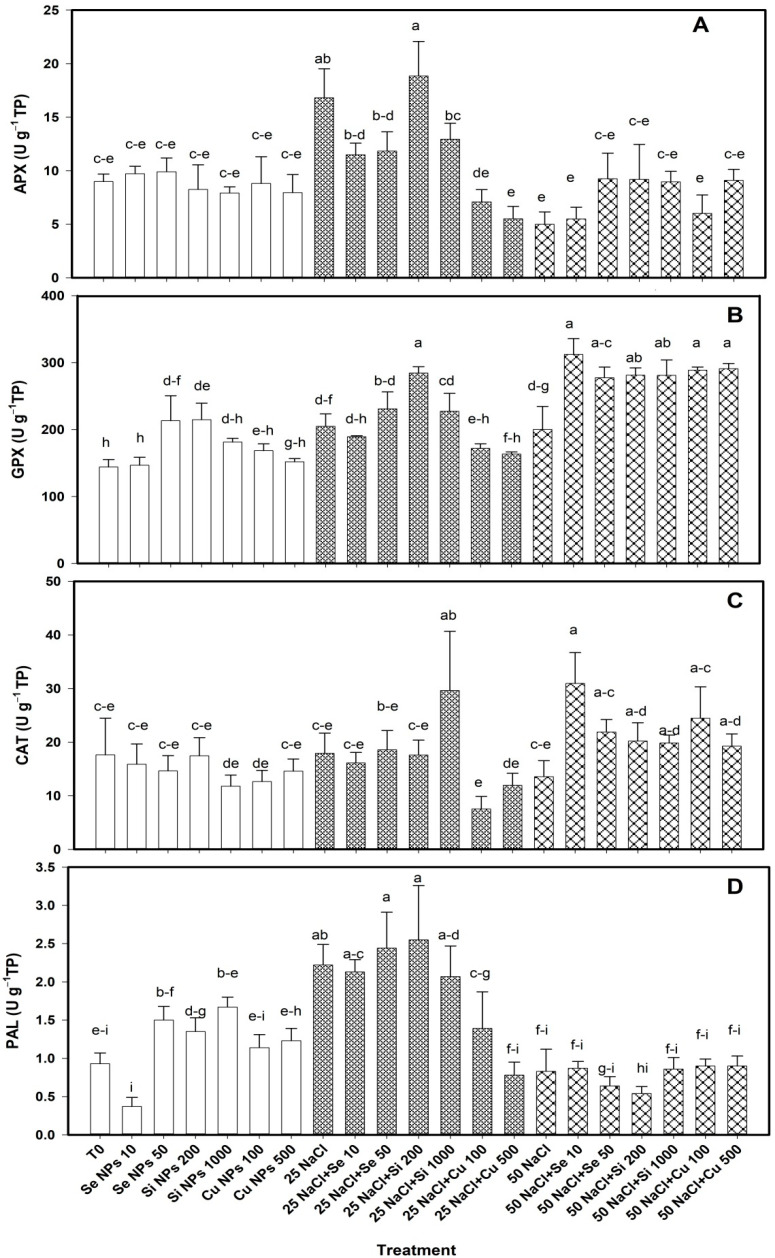
Enzymatic activity of ascorbate peroxidase (**A**), glutathione peroxidase (**B**), catalase (**C**) and phenylalanine ammonia lyase (**D**) in leaves of bell pepper plants under saline stress. T0: control; 25 NaCl: 25 mM NaCl; 50 mM NaCl: 50 mM NaCl; NPs: nanoparticles; Numbers represent de quantities of NaCl (mM) or NPs (mg L^−1^) applied. Different letters indicate significant differences between treatments according to Least Significant Difference Fisher´s Test (α = 0.05). n = 5 ± standard error.

The activity of the enzymes was modified with the application of nanoparticles (Figure 3). When the plants were not subjected to saline stress, the treatments did not impact on the APX activity (Figure 3A). At 25 mM NaCl, both doses of Cu NPs decreased the activity of this enzyme by 59–67% with respect to its control. At 50 mM there were no differences between treatments.

In the absence of saline stress, GPX activity was increased with the application of the Si 200 and Se 50 treatments by 49% and 48% respectively compared to T0. At 25 mM NaCl, only the Si 200 treatment increased the activity of this enzyme by 39% compared to its control. At 50 mM, all treatments increased GPX activity (Se 10 ≥ Cu 100 ≥ Cu 500 ≥ Si 200 ≥ Si 100 ≥ Se 50) in a range of 39–56% with respect to their control (Figure 3B).

CAT activity was not modified with the application of nanoparticles when there was no saline stress in the plants (Figure 3C). At 25 mM, only the Si 1000 treatment increased CAT activity by 65% compared to its control. At 50 mM NaCl, only the Se 10 treatment increased CAT activity by 128% compared to its control.

PAL activity was not modified with the application of nanoparticles when the plants were not subjected to saline stress (Figure 3D). At 25 mM NaCl, the single application of salinity increased PAL activity with respect to T0 (139%); however, the application of both doses of Cu NPs decreased the activity of this enzyme by 28–65% compared to its control. At 50 mM no differences were observed between treatments.

**Figure 4:**
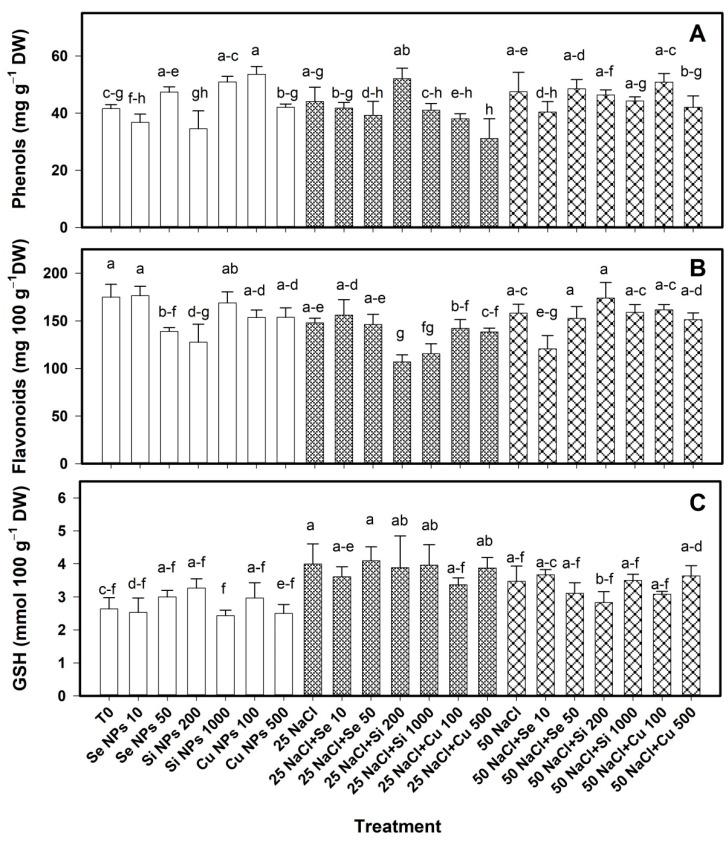
Content of phenols (**A**), flavonoids (**B**) and glutathione (**C**) in leaves of bell pepper plants under saline stress. T0: control; 25 NaCl: 25 mM NaCl; 50 mM NaCl: 50 mM NaCl; NPs: nanoparticles; Numbers represent de quantities of NaCl (mM) or NPs (mg L^−1^) applied. Different letters indicate significant differences between treatments according to Least Significant Difference Fisher´s Test (α = 0.05). n = 5 ± standard error.

The content of non-enzymatic antioxidants in the leaves was modified by the application of the nanoparticles (Figure 4). In the absence of saline stress, the content of phenols increased with the Cu 100 treatment by 29% with respect to T0. At 25 mM NaCl, no treatment increased the phenol content; on the contrary, the Cu 500 treatment caused a 29% decrease with respect to its control. At 50 mM NaCl, no differences were observed between treatments (Figure 4A).

The flavonoid content decreased with the Se 50 and Si 200 treatments of 20% and 27% respectively compared to T0 (Figure 4B). At 25 mM NaCl, both doses of Si NPs, 200 and 1000 mg L^−1^, caused a decrease of 28% and 22% respectively compared to their control. At 50 mM NaCl, Se 10 treatment caused a 24% decrease with respect to its control.

In the absence of saline stress, GSH was not modified by the application of nanoparticles (Figure 4C). At 25 mM NaCl, only the application of salinity increased the GSH content by 51% with respect to T0; however, nanoparticle treatments had no effect on this condition. At 50 mM NaCl there was no effect of the treatments applied on the content of this compound.

### 2.3. Bioactive Compounds in Pepper Fruits

**Figure 5:**
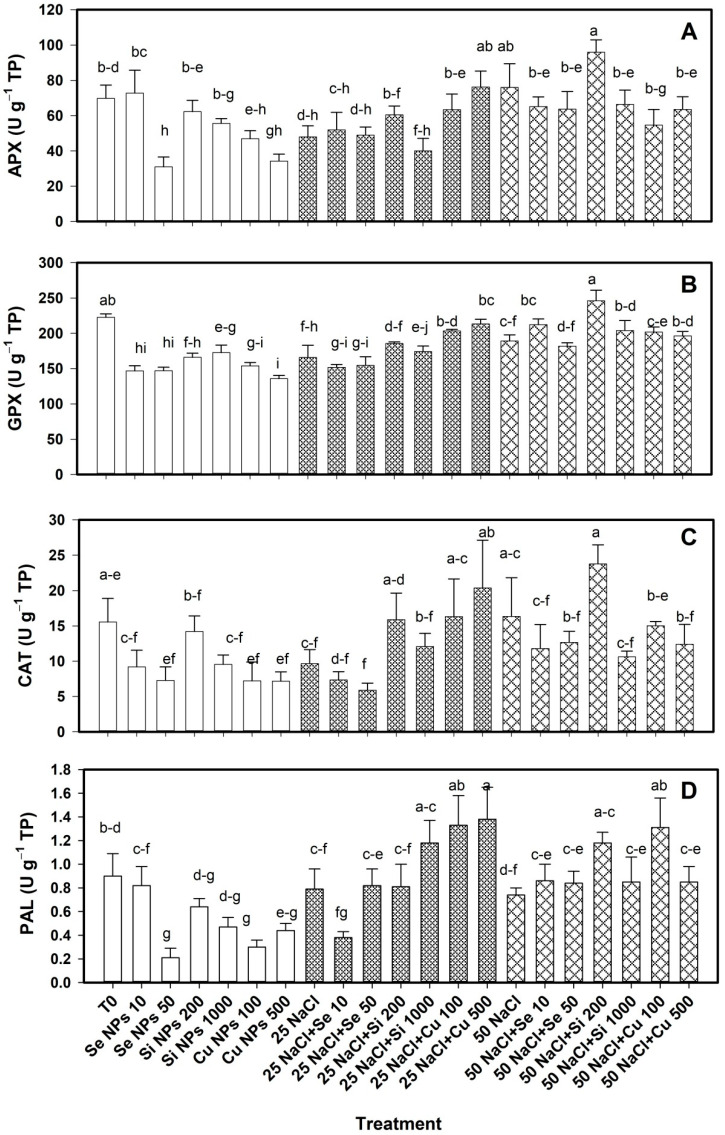
Enzymatic activity of ascorbate peroxidase (**A**), glutathione peroxidase (**B**), catalase (**C**) and phenylalanine ammonia lyase (**D**) in bell pepper fruits of plants under saline stress. T0: control; 25 NaCl: 25 mM NaCl; 50 mM NaCl: 50 mM NaCl; NPs: nanoparticles; Numbers represent de quantities of NaCl (mM) or NPs (mg L^−1^) applied. Different letters indicate significant differences between treatments according to Least Significant Difference Fisher´s Test (α = 0.05). n = 5 ± standard error.

The enzymatic activity in the bell pepper fruits showed differences between treatments in APX, GPX, CAT and PAL (Figure 5). In the absence of salinity, the Se 50 treatments and both doses of Cu NPs decreased the APX activity compared to the control in a range of 33–56% (Figure 5A). However, at 25 mM NaCl, Cu 500 treatment induced an increase in APX activity, 59% more than their respective control. At 50 mM NaCl the treatments had no effect on the APX activity.

The GPX activity decreased with all the applied treatments when the plants did not have saline stress (Figure 5B). However, at 25 mM NaCl, both doses of Cu NPs increased GPX activity by 23–28% compared to its control. While at 50 mM, only the Si NPs at 200 mg L^−1^ increased GPX activity by 30% compared to its control.

No treatment had an effect on catalase activity in the absence of saline stress (Figure 5C). At 25 mM NaCl, only the Cu NPs at 500 mg L^−1^ increased the catalase activity by 112% with respect to their control. At 50 mM NaCl the treatments had no effect on the activity of this enzyme.

In the absence of saline stress, PAL activity decreased by Se 50 treatments and both doses of Cu NPs by 51–77% compared to T0 (Figure 5D). At 25 mM NaCl, PAL activity increased with both doses of Cu NPs in a range of 68–75% compared to its control. While at 50 mM NaCl, PAL activity increased with the Si 200 and Cu 100 treatments by 59% and 77% respectively compared to their control.

**Figure 6:**
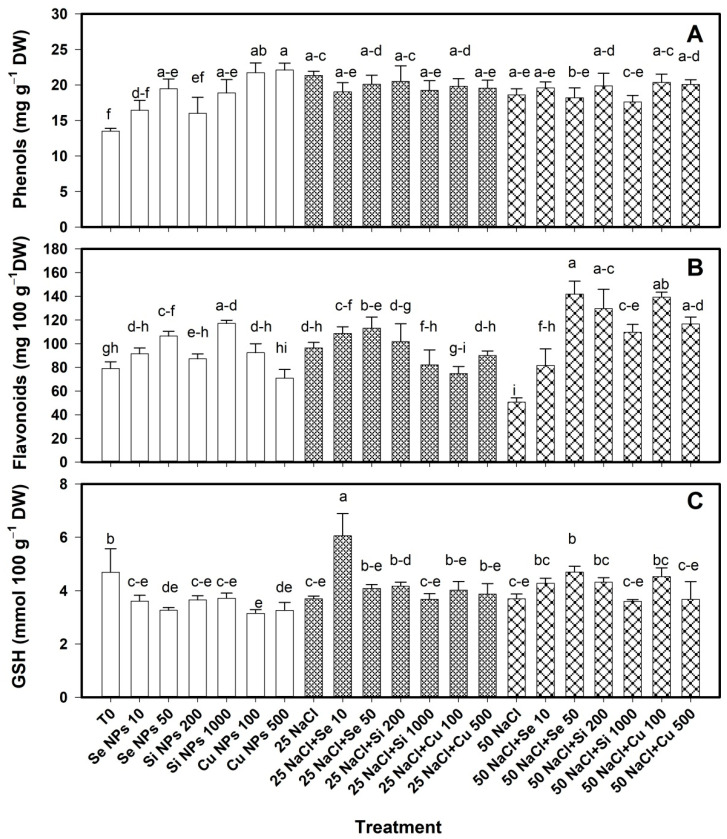
Content of phenols (**A**), flavonoids (**B**) and glutathione (**C**) in bell pepper fruits of plants under saline stress. T0: control; 25 NaCl: 25 mM NaCl; 50 mM NaCl: 50 mM NaCl; NPs: nanoparticles; Numbers represent de quantities of NaCl (mM) or NPs (mg L^−1^) applied. Different letters indicate significant differences between treatments according to Least Significant Difference Fisher´s Test (α = 0.05). n = 5 ± standard error.

The content of phenols, flavonoids and glutathione in bell pepper fruits was modified by the application of nanoparticles (Figure 6). In the absence of saline stress, the Cu 500 and Cu 100 treatments increased the content of phenols in the fruit by 65% and 61% with respect to T0. Furthermore, the Se 50 and Si 1000 treatments also increased the content of this compound by 44% and 40% respectively. At 25 and 50 mM NaCl the nanoparticle treatments had no effect on the phenol content (Figure 6A).

The flavonoid content in the absence of saline stress increased with the Si 1000 and Se 50 treatments by 48% and 35% respectively compared to T0 (Figure 6B). At 25 mM NaCl, the treatments did not differ from each other. At 50 mM all the nanoparticle treatments increased the flavonoid content, especially Cu 100 and Si 200 with 175% and 156% more than their control.

The GSH content in the fruit decreased with all nanoparticle treatments (Figure 6C). However, at 25 mM NaCl, only Se 10 treatment increased the GSH content by 64% with respect to its control. At 50 mM NaCl, only GSH was increased with the Se 50 treatment by 27% compared to its control.

**Figure 7:**
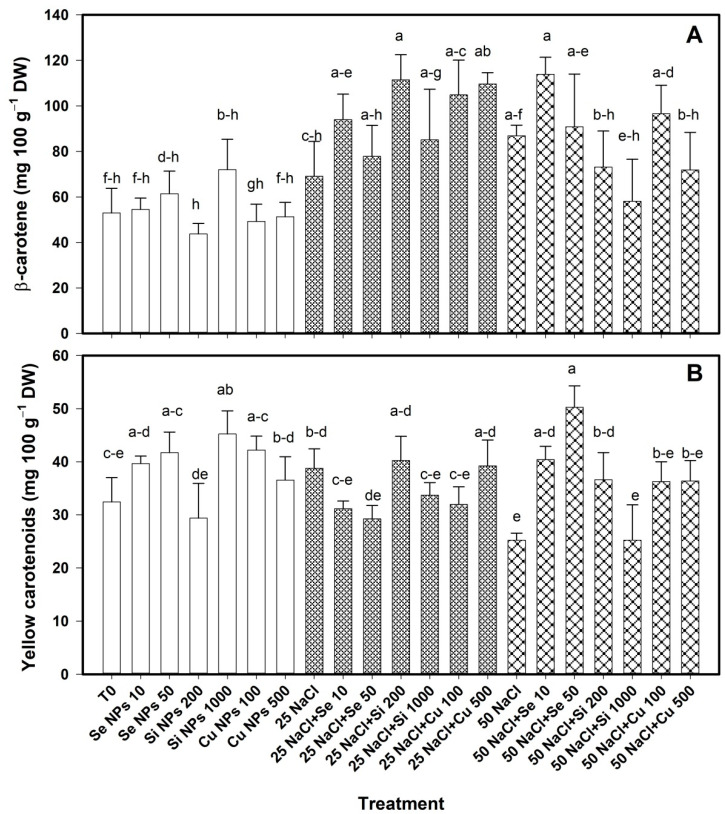
Content of β-carotene (**A**) and yellow carotenoids (**B**) in bell pepper fruits of plants under saline stress. T0: control; 25 NaCl: 25 mM NaCl; 50 mM NaCl: 50 mM NaCl; NPs: nanoparticles; Numbers represent de quantities of NaCl (mM) or NPs (mg L^−1^) applied. Different letters indicate significant differences between treatments according to Least Significant Difference Fisher´s Test (α = 0.05). n = 5 ± standard error.

The carotenoid content in the bell pepper fruit was modified with the application of nanoparticles (Figure 7). The β-carotene in the absence of saline stress was not modified by the application of nanoparticles (Figure 7A). At 25 mM NaCl, the Si 200 and Cu 500 treatments increased the content of this compound by 61% and 59% respectively compared to their control. At 50 mM NaCl there were no differences between treatments.

In the absence of salinity, the yellow carotenoid content increased by 40% with the Si 1000 treatment compared to T0. At 25 mM NaCl there were no differences between treatments. At 50 mM NaCl, the Se 50, Se 10 and Si 200 treatments increased the content of yellow carotenoids by 99%, 60%, and 45% respectively compared to their control (Figure 7B).

## 3. Discussion

Salinity is an abiotic stress that, among other things, reduces the plant’s ability to absorb water, causes senescence, reduces the photosynthetic area of the leaves, which reduces the growth rate [22]. In addition, it induces osmotic stress, and nutrient imbalance due to the effects of toxic Na^+^ ions [23]. All these effects modify not only the yield of the fruit, but also the content of bioactive compounds.

Houimli et al., [24] reported a reduction of 15.6% in chlorophyll *a*, and 39.2% in chlorophyll *b* in pepper under saline stress. The decrease in chlorophyll content when the plants were not subjected to saline stress is attributed to the fact that nanoparticles can generate oxidative stress depending on their characteristics and the cultured species, and induce peroxidation of the chloroplast membrane and degradation of the photosynthetic pigments [25]. By subjecting the plants to saline stress, osmotic stress is also generated, which limits water access to the plants, which leads to loss of turgor and cell dehydration, in addition to causing ionic toxicity and nutritional imbalance and increasing generation excessive ROS, which are potentially toxic and harmful radicals capable of causing oxidative damage to proteins, DNA, and lipids [7]. However, NPs have the ability to increase the content of chlorophylls, which results in a decrease in ROS levels, and a greater photochemical efficiency [10]. In addition, NPs have a positive effect on some chloroplast enzymes that are involved in the biosynthesis of photosynthetic pigments [14]. This can result in the increase of chlorophylls or other accessory pigments in the leaves of the plants. This is particularly important since accessory pigments such as carotenoids are important antioxidants because they are important agents in dissipating excess excitation energy in the PSII antenna, especially under stress conditions [26]. Morales-Espinoza et al. [27] reported an increase in chlorophylls *a*, *b* and total of 71%, 120% and 97% respectively when 20 mg L^−1^ of Se NPs were applied in tomato plants stressed with 50 mM NaCl. Hussein et al. [14] reported that the application of Se NPs induced a higher chlorophyll content in peanut crop. Likewise, when applying SiO_2_ NPs in strawberry developed under saline stress, the chlorophyll content increased [28]. Application of 100 mg L^−1^ TiO_2_ in *Dracocephalum moldavica* L. increased the content of chlorophyll *a*, *b*, and carotenoids when there was no salinity stress; however, under salinity conditions, it was able to increase the pigment content, managing to reverse the negative effect of salinity [29]. The application of Cu NPs (2.5 mg L^−1^) in *Oryza sativa* increased by 50% the content of carotenoids (β-carotene, violaxanthin and lutein), however in higher doses the amount of carotenoids decreased [30]. In strawberry crop, the carotenoid content was increased with the application of SiO_2_ NPs [28]. Ghadakchi asl et al. [31] reported that the content of carotenoids in grapes under saline stress conditions increased, and at Si NPs (2 mM) the content of these metabolites also increased.

NPs are known to increase the activity of ROS-scavenging antioxidant enzymes, mainly ascorbate peroxidase (APX), superoxide dismutase (SOD), catalase (CAT), monodehydroascorbate reductase (MDHAR), glutathione reductase (DHAR) to protect cells against oxidative damage [32]. However, they also increase the content of non-enzymatic antioxidant compounds such as flavonoids and phenols [33]. This is due to the biostimulation capacity of NPs and nanomaterials in general, derived from the unique characteristics of these compounds [34]. The physical characteristics of the NPs i.e., size, shape, porosity, etc. determine the characteristics of charges and free energy of their surface, which is of utmost importance since the first interaction between plant cells and NPs occurs at the surface level, this causes a series of responses at the cellular level such as production of stress biomarkers that induces the activation of defense systems [35]. Ultimately this results in the production of the non-enzymatic antioxidants and antioxidant enzymes. The increase in the activity of antioxidant enzymes can increase tolerance to salt stress due to the decrease in oxidative damage in the membranes [36]. Siddiqui et al. [10] reported that the application of Si NPs increased the activity of CAT and APX in *Cucurbita pepo* L. under conditions of saline stress (120 mM NaCl). Gohari et al. [29] demonstrated that the application of TiO_2_ NPs in *Dracocephalum moldavica* L. managed to reduce the negative effects of salinity, due to the increased activity of antioxidant enzymes such as guaiacol peroxidase (GP), APX, GPX and SOD, which resulted in a more efficient ROS detoxification. In fruits specifically, the activity of these enzymes is essential, since it keeps ROS levels, such as H_2_O_2_, low and prevents damage to cells due to lipid peroxidation and membrane leakage; this in turn maintains the quality of the fruits and the content of non-enzymatic antioxidant compounds such as ascorbic acid and glutathione [37].

PAL is very important because it participates in the biosynthesis of phenols, by producing trans-cinnamic acid skeletons from phenylalanine [38]. For this reason, the activity of this enzyme can influence the activity of other non-enzymatic compounds. As observed in this work, it has been consistently reported that the application of different types of NPs can increase the content of bioactive compounds in fruits. Cumplido-Nájera et al. [39] reported that the application of Cu NPs+potassium silicate increased PAL activity both in leaves and fruits of tomato plants. The application of Cu NPs-chitosan-PVA increased the content of phenols in tomato leaves under conditions of saline stress [20]. Also, Hernández-Fuentes et al. [40] reported that the application of Cu NPs in tomato plants under saline stress increased the content of phenols in the fruits. Morales-Espinoza et al. [27] showed that when applying Se NPs in tomato crops under salinity stress, the content of flavonoids, lycopene and β-carotene in tomato fruits was increased. These same authors reported an increase in the activity of antioxidant enzymes in tomato fruits, especially APX, CAT and SOD, which participate in the detoxification of ROS and prevent lipid peroxidation of the membranes. Additionally, the application of Cu NPs in the tomato crop under salinity increased the content of bioactive compounds in tomato fruits, mainly vitamin C and lycopene, as well as the activity of the PAL, APX, GPX and CAT enzymes [41].

Bioactive compounds such as ascorbic acid, phenols, flavonoids and carotenoids are among the most important plant antioxidants [42]. Ultimately, the antioxidant capacity will be given by hydrophilic and lipophilic compounds. The lipophilic ones are mainly carotenoids and vitamin E [43], while the hydrophilic ones are vitamin C, GSH, phenols and flavonoids [42,44]. However, its antioxidant activity is related to potential health functionality against various chronic non-communicable diseases [45]. For this reason, the consumption of this type of food, rich in bioactive compounds, is very important.

## 4. Materials and Methods

### 4.1. Establishment of the Experiment

The experiment was established in a greenhouse with a polyethylene cover and natural ventilation for temperature control. Seeds of F1 hybrid yellow pepper (*Capsicum annuum* L.), variety “Kitrino” (Seminis, Oxnard, CA, USA) were used. The transplant was carried out in 12 L polyethylene bags using a mixture of peat and perlite in a 1:1 ratio as substrate. The nutrition of the seedlings was carried out with the application of Steiner solution [46] through a directed irrigation system. The cultivation was developed with two stems with Dutch type tutored and a planting density of 2.5 plants per square meter, during 170 days from the transplant.

### 4.2. Application of Treatments

The experiment consisted of three salinity conditions (No NaCl, 25 mM NaCl and 50 mM NaCl), and six nanoparticle treatments (Se NPs at 10 and 50 mg L^−1^ [Se 10, Se 50], SiO_2_ NPs at 200 and 1000 mg L^−1^ [Si 200, Si 1000], Cu NPs at 100 and 500 mg L^−1^ [Cu 100, Cu 500]) plus a control without application. The doses and types of NPs, as well as the application intervals, were selected based on the results presented in in previous works [27,39,41,47]. Saline stress was induced by the application of NaCl at 25 mM and 50 mM in the nutrient solution throughout the culture cycle, starting three days after transplantation. The nanoparticle treatments were applied by drenching 10 mL for each plant of a solution that contained the different concentrations and types of nanoparticles. A total of five applications were made at 30-day intervals starting from the transplant.

The Se NPs and Cu NPs used are of spherical morphology and with a size of 2–20 nm and 50 nm respectively, the complete characterization is described in [47]. SiO_2_ NPs have a spherical morphology, size 10–20 nm, a surface area of 160 m^2^ g^−1^ and an apparent density of 0.08–0.1 g cm^−3^ (SkySpring Nanomaterials Inc., Houston, TX, USA), the full characterization is described in [48].

### 4.3. Biochemical Analysis

For the biochemical analyzes, the leaf and fruit samples were taken 100 days after transplantation. The fruits were selected when they were fully mature. And the selected leaves were the closest to the fruits collected for the analyzes. The samples were collected on ice and frozen at −20 °C to be lyophilized later.

#### 4.3.1. Photosynthetic Pigments

The chlorophyll, Lycopene and β-carotene content (mg 100 g^−1^ dry weight [DW]) was determined according to the method of Nagata and Yamashita [49]. Yellow carotenoids (β-carotene, β-cryptoxanthin, zeaxanthin) were evaluated according to the method reported by [50]. The measurements of yellow carotenoids were expressed as milligrams per 100 g of dry weight (mg 100 g^−1^ DW).

#### 4.3.2. Enzymatic Activity

Ascorbate peroxidase (EC 1.11.1.11) was determined by the method of Nakano and Asada [51] and is expressed as U per gram of total proteins (U g^−1^ TP), where U is equal to the μmol of oxidized ascorbate per milliliter per minute. Glutathione peroxidase (EC 1.11.1.9) (U per gram of total proteins (U TP^−1^), where U is equal to the mM equivalent of reduced glutathione (GSH) per milliliter per minute), was determined by the method of Flohé and Günzler [52,53]. Catalase (EC 1.11.1.6) (U TP^−1^, where U is equal to the mM equivalent of H_2_O_2_ consumed per milliliter per minute) was quantified by the method of Dhindsa et al. [54]. Phenylalanine ammonia lyase (PAL) (EC 4.3.1.5) was determined according to the method of Sykłowska-Baranek et al. [55].

#### 4.3.3. Non-Enzymatic Antioxidant Compounds

Phenols (mg g^−1^ DW) were determined by the method of Singleton et al. [56] using the Folin–Ciocalteu reagent. Flavonoids (mg 100 g^−1^ DW) were determined following the method described by Arvouet-Grand et al. [57]. The glutathione (GSH) content (mmol 100 g^−1^ DW) was determined following the method described in Xue et al. [52].

### 4.4. Statistical Analysis

The experiment was established in a completely randomized design with a factorial arrangement (3 × 7), totaling 21 treatments. Five replicates per treatment were analyzed and an analysis of variance and a Fisher’s least significant difference (LSD) mean comparison test (α = 0.05) were performed in the Infostat software (2020v).

## 5. Conclusions

Salt stress negatively affects the content of chlorophylls and accessory pigments (lycopene, and β-carotene) in the leaves of bell pepper plants, which can affect the normal development of plants. In fruits, salinity also has a negative impact, since it reduces the content of important bioactive compounds such as flavonoids and glutathione.

The application of the different nanoparticles (selenium, silicon and copper) showed positive results, since it increased the content of chlorophylls, lycopene and GPX activity in the leaves. And in the fruits, the application of nanoparticles induced positive effects in most of the bioactive compounds such as phenols, flavonoids, GSH, β-carotene and yellow carotenoids. Moreover, these NPs increased APX, GPX, CAT and PAL activity, which are essential for the detoxification of ROS and avoid lipid peroxidation damage, thus maintaining the content of bioactive compounds and the quality of the fruits.

The application of nanotechnology in agriculture represents enormous opportunities to improve this activity that is fundamental for humanity. However, more information is still lacking about the implications that nanoparticles may have on the agroecosystem due to trophic chains, as well as the possible impacts on human health. Although, as has been shown in this study and others, the immediate results in the quality of the fruits and crops are positive, knowledge about this area must be deepened in order for its application to be safe. Fortunately, there are novel methods that can help to understand the functioning of nanoparticles in crops and their impacts in the medium and long term. These include the application of artificial intelligence, optimization algorithms, and artificial neural network models [58,59,60,61,62] that can be excellent tools for the study of nanotechnology in agriculture and in fruit quality.
